# Factors that affect substance users’ suicidal behavior: a view from the Addiction Severity Index in Korea

**DOI:** 10.1186/1744-859X-12-35

**Published:** 2013-11-12

**Authors:** Min Kwon, Soo Yang, Kyongran Park, Dai-Jin Kim

**Affiliations:** 1 Addiction Research Institute, Department of Psychiatry, Seoul St. Mary’s Hospital, The Catholic University of Korea, Seoul, South Korea; 2 College of Nursing, The Catholic University of Korea, Seoul, South Korea; 3 Department of Psychiatry, Seoul St. Mary’s Hospital, College of Medicine, The Catholic University of Korea, 222 Banpo-daero, Seocho-gu, Seoul 137-701, South Korea

**Keywords:** Substance use, Suicidal behavior, Suicidal ideation, Suicide attempts

## Abstract

**Background:**

In South Korea, it has not been easy to negotiate studies that target drug users who are being punished by law, and accordingly, no study on suicidal ideation among substance users has been accomplished yet. In this study, the factors that affect substance users’ suicidal ideation were confirmed.

**Methods:**

It was based on the data collected from 'The 2009 Study on Substance-Dependent Individuals in Korea’ , which was conducted by The Catholic University of Korea in 2010 as a project sponsored by the Ministry of Health and Welfare of Korea. This study targeted 523 former hospital inpatients, prison inmates, and persons under protective supervision who had used substances such as psychotropic drugs, marijuana, and narcotic agents, and were in the recovery stage at various treatment/rehabilitation centers. Student’s *t* and chi-square tests were used, and multivariate analysis was performed to examine the strength of the relationships between suicide ideation and various factors.

**Results:**

According to this study, 41% of these substance users planned suicide with suicidal ideation. Suicidal ideation was confirmed as associated with an unsatisfactory domestic environment, insufficient and unsatisfactory spare time experiences with others, emotional abuse, severe depression, and trouble with controlling violent behavior. Of the substance users who had planned to commit suicide, 56% attempted suicide. Their suicide attempts were shown to have been associated with insufficient protective supervision and the experiences of physical abuse, trouble with controlling violent behavior, and doctors’ prescriptions due to psychological or emotional problems.

**Conclusion:**

Based on this analysis of the factors that affect suicidal behavior, preventive measures and strategies for substance user were suggested in this study.

## Background

According to the annual report on 2010 death causes in Korea published by Statistics Korea, suicide is the fourth common cause of death among both men and women, after cancer, cerebrovascular diseases, and heart diseases, with incidences of 41.4 in 100,000 men and 21.0 in 100,000 women [1]. Among the member countries of the Organization for Economic Cooperation and Development, Korea reported the highest suicide rate [2]. While other developed countries have reported a gradual decrease in their suicide rate, Korea has reported the opposite trend, which has been recognized as a critical national issue.

Meanwhile, the association between suicide and substance use has been continuously reported and evaluated through psychological autopsies and studies on the suicide rate among people with specific diseases [3]. In particular, in a study on substance use disorders that had caused a suicide rate increase, the incidence of suicide among heroin-dependent patients was reported as 20 times higher than that of the general population [4]. In a study on suicides in New York City, the suicide rate among cocaine users was reported as one in every five citizens [5]. Serious deterioration of human relations of substance users was reported as a common cause of suicide attempts, and suicidal ideation frequently developed in substance abuse patients who experienced emotional abuses in their childhood [6]. The relationship between substance use disorders and mood disorders, and the association of resistance to psychological trauma treatments have been explained [7].

Nevertheless, these studies have reported that comorbidity such as mood and personality disorders was caused by repeated or mixed substance use, so specific factors that affect suicidal ideation of substance users have not been clearly investigated yet. In Korea, it has not been easy to negotiate studies that target drug users who are being punished by law, and accordingly, no study on suicidal ideation among substance users has been accomplished yet.

To confirm the characteristics of substance users, the Addiction Severity Index (ASI) was used in this study. With the interviews that were designed by experts, data were collected and confirmed. ASI is a semi-structured clinical research interview that is widely used in substance abuse treatment settings in the USA and many other countries. This instrument was designed to assess the problem severity in seven functional domains: Medical Information, Employment, Drug/Alcohol Use, and the Legal, Family, Social, and Psychological aspects [8]. In this study, the factors that affected the suicidal behavior of drug users were analyzed, and preventive measures, strategies and treatment programs were suggested.

## Methods

### Participants

This study targeted 523 former hospital inpatients, prison inmates, and persons under protective supervision who had used substances such as psychotropic drugs, marijuana, and narcotic agents, and were in the recovery stage at various treatment/rehabilitation centers. The subjects agreed to participate in this study with the permission of their centers. The interviewers explained to them the nature of this study before the ASI interviews, and their written consent to receive information on the purpose, process, and time of this study, and to surrender their privacy was obtained. The survey was conducted from March 2009 to February 2010. The mean age of the subjects was 43.31 (standard deviation = 8.38 and range = 16–70 years). There were 489 males (93.5%).

### Procedure

In this study, the factors that affected the drug users’ suicidal ideation were confirmed based on the data collected from the 2009 Study on Substance-Dependent Individuals in Korea, which was conducted by The Catholic University of Korea in 2010 as a project sponsored by the Ministry of Health and Welfare. The data were collected from anonymous volunteer participants, and no compensation was offered to the subjects with respect to this study. To secure the anonymity of the subjects, the data that were mainly on ASI were inputted as codes in Excel files.

### Questionnaires

The Korean version of the ASI was used in the present study [9]. It elicits the respondent’s self-reported problems in seven areas: Medical Information, Employment, Drug/Alcohol Use, and Legal, Family, Social, and Psychological aspects. The Medical Status domain gathers basic information about medical history. It addresses information about lifetime hospitalizations, long-term medical problems, and recent physical ailments. The Employment/Support Status domain gathers basic information about work experience and current sources of income. The Drug/Alcohol Use domain gathers basic information about the patient’s substance abuse history. It addresses information about current and lifetime substance abuse, the consequences of abuse, periods of abstinence, treatment episodes, and the financial burden of substance abuse. The Legal Status domain gathers basic information about the patient’s legal history. It addresses information about probation or parole, legal charges, convictions, incarcerations or detainments, and illegal activities. The Family/Social Relationship domain assesses relationship problems with family members or friends. The Psychiatric Status domain is used not to diagnose psychiatric disorders but to assess the experience of various psychiatric symptoms other than those associated with the effects of alcohol or drugs [10].

The ASI was administered by two psychologists who were experts in drug dependence, carefully read the ASI manual, and learned the interview methods themselves. The average time required for interview of the questionnaire was 40 min.

### Statistical analysis

For the statistical analysis, Student’s *t* and chi-square tests were used. The odds ratios and the 95% confidence intervals (CIs) were also calculated. To examine the strength of the relationships between suicide ideation and various factors, multivariate analysis was used. All the analyses were conducted using PASW statistics (version 18.0).

### Ethics and approvals

This study was conducted after the written consent of the anonymous volunteer participants was obtained. The ethical standards in the 1964 Declaration of Helsinki were followed. This study was approved by the local ethics committee (reference KC13EISI0233).

## Results

### Comparison of the characteristics of substance users with and without suicidal ideation

Of the 523 substance users, 215 (41.1%) planned suicide with suicidal ideation. Their mean age was 42, and 201 (93.5%) of them were male. A statistically significant difference in age was observed between the groups with and without suicidal ideation, as the mean age of the group without suicidal ideation was 44, higher than that of the other group.

Among the items in the ASI, a statistically significant difference in the detoxification treatment experience was observed between the groups with and without suicidal ideation (*p* = 0.001). The frequency of detoxification treatments in the group with suicidal ideation was 39 (18.2%), which was higher than that in the other group. In the Social Information domain, statistically significant differences were observed between the groups according to the marital status; satisfaction level with the family environment; spare time experiences with others; satisfaction with such spare time experiences; serious family problems; experiences of emotional, physical, and sexual abuse; conflicts with family members; human relations problems; and recognition of the need for the treatments. The group with suicidal ideation showed a lower satisfaction level with their family environment; they spent most of their spare time alone, and their satisfaction level with their spare time was also relatively low. They had comparatively more serious problems in their relations with their parents, siblings, and friends, and had more experiences of emotional, physical, and sexual abuse than the group without suicidal ideation.

In the Psychological Information domain, the group with suicidal ideation reported more serious depression, anxiety, tension, and hallucinations. They also experienced more difficulty in understanding, concentration, and memorization, and their control over violent behavior was worse than that of the group without suicidal ideation. The group with suicidal ideation also more often received doctors’ prescriptions due to psychological and emotional problems. They suffered more pain due to such problems and recognized more the need for treatments than the group without suicidal ideation (Table 1).

**Table 1:** Characteristics of substance users with and without suicidal ideation

**Characteristics**	**With suicidal ideation**	**Without suicidal ideation**	***P *** **value**
	**(*N*** **= 215)**	**(*N*** **= 308)**	
General information			
Age in years	42.09 ± 8.08	44.21 ± 8.48	0.004
Male	201 (93.5)	287 (93.5)	0.999
Education (grades)	10.01 ± 2.24	10.27 ± 2.34	0.208
Employed	43 (20.0)	68 (22.2)	0.588
Drug and alcohol information			
Detoxification treatment	39 (18.2)	25 (8.2)	0.001
Social information			
Marital status			0.013
Married	62 (28.8)	122 (39.7)	
Single	84 (39.1)	87 (28.3)	
Separated/divorced/widowed	69 (32.1)	98 (31.9)	
Satisfaction of family environment	129 (60.8)	229 (74.6)	0.001
Satisfaction of spare time	135 (63.1)	248 (81.0)	<0.001
Spare time			<0.001
with others	125 (59.0)	236 (77.1)	
alone	87 (41.0)	70 (22.9)	
Serious family problem			
with mother	78 (36.3)	69 (22.5)	0.001
with father	81 (37.7)	69 (22.5)	<0.001
with brothers and sisters	66 (30.8)	49 (16.0)	<0.001
with friend	86 (40.0)	69 (22.5)	<0.001
Emotional abuse	107 (50.0)	60 (19.5)	<0.001
Physical abuse	34 (15.9)	17 (5.5)	<0.001
Sexual abuse	5 (2.3)	0 (0.0)	0.007
Pain from family problem	0.99 ± 1.47	0.58 ± 1.26	0.001
Pain from social problem	0.84 ± 1.36	0.49 ± 1.13	0.002
Need for treatment for family problem	0.80 ± 1.35	0.46 ± 1.15	0.003
Need for treatment for social problem	0.64 ± 1.23	0.41 ± 1.04	0.024
Psychological information			
Serious depression	136 (63.3)	56 (18.2)	<0.001
Serious anxiety or tension	110 (51.2)	40 (13.0)	<0.001
Hallucinations	44 (20.5)	10 (3.3)	<0.001
Trouble with understanding, concentrating, or remembering	111 (51.6)	62 (20.3)	<0.001
Trouble with controlling violent behavior	115 (53.5)	59 (19.2)	<0.001
Prescription experience	81 (37.7)	40 (13.0)	<0.001
Pain from psychological problem	2.61 ± 1.43	1.24 ± 1.53	<0.001
Need for treatment	2.35 ± 1.66	0.99 ± 1.46	<0.001

The categorical variables are presented as *n* (%) in the chi-square test, and the continuous variables are presented as the mean ± SD in the Student’s *t* test.

### Analysis of the factors that affect the suicidal ideation of substance users

The factors that affect suicidal ideation were analyzed with the 215 subjects with suicidal ideation. In the logistic regression, the suicidal ideation of the subjects was shown to have been associated with their dissatisfaction with their domestic environment, which had an odds ratio (OR) of 0.50, less spare time experiences with others (OR, 0.37), dissatisfaction with their spare time experiences (OR, 0.54), experiences of emotional abuse (OR, 1.87), experiences of serious depression (OR, 2.01), and trouble with controlling violent behavior (OR, 2.59) (Table 2).

**Table 2:** Logistic stepwise regression analysis of substance users with suicidal ideation

**Category**	**Odds ratio**	**95% CI**	***P *** **value**
Satisfaction of family environment	0.500	0.306–0.818	0.006
Spare time with another	0.366	0.220–0.611	<0.001
Satisfaction of spare time	0.538	0.315–0.919	0.023
Emotional abuse	1.870	1.085–3.223	0.024
Serious depression	2.006	1.118–3.599	0.020
Trouble with controlling violent behavior	2.587	1.559–4.293	<0.001

### Comparison of the characteristics of substance users with and without suicide attempts

Of the 215 substance users who had suicidal ideation and planned to commit suicide, 120 (55.8%) attempted suicide. Their mean age was 42, and 112 (93.3%) of them were male. No statistically significant difference between the general characteristics of the suicide attempts group and the no-suicide attempts group was observed.

The comparison of the characteristics in the Drug and Alcohol Information domain of the ASI according to the suicide attempts showed that the suicide attempts group reported 23 (19.2%) cases of delirium tremens and 74 (61.7%) cases of drug overdose, which were significantly more than in the no-suicide attempts group (*p* = 0.017 and *p* = 0.019, respectively). In the Medical Information domain, the suicide attempts group had more medical problems. In the Legal Information domain, statistically significant differences between the suicide attempts group and the no-suicide attempts group were observed according to the status of their protective supervision, their criminal record with assault, the period of their imprisonment, and the severity of their legal problem. The suicide attempts group received less protective supervision and had fewer criminal records with assault, longer imprisonment periods, and more severe legal problems than the no-suicide attempts group.

In the Social Information domain, 24 subjects (20.0%) in the suicide attempts group experienced physical abuse, which was significantly more than the nine subjects (9.6%) in the no-suicide attempts group. In the Psychological Information domain, the suicide attempts group experienced more serious depression and trouble with controlling their violent behavior, and received more doctors’ prescriptions than the no-suicide attempts group (Table 3).

**Table 3:** Characteristics of substance users with and without suicide attempts

**Characteristics**	**With suicide attempts**	**Without suicide attempts**	***P *** **value**
	**(*N*** **= 120)**	**(*N*** **= 95)**	
General information			
Age in years	41.55 ± 7.60	42.52 ± 8.62	0.384
Male	112 (93.3)	89 (93.7)	0.918
Education (grades)	9.78 ± 2.41	10.28 ± 2.01	0.100
Employed	24 (20.0)	19 (20.0)	1.000
Drug and alcohol information			
Delirium tremens	23 (19.2)	7 (7.4)	0.017
Drug overdose	74 (61.7)	42 (44.7)	0.019
Medical information			
Pain from medical problem	0.99 ± 1.41	0.61 ± 1.09	0.027
Legal information			
Protective supervision	27 (22.5)	36 (37.9)	0.016
Criminal record with assault	64 (53.3)	34 (35.8)	0.013
Period of imprisonment	81.09 ± 69.36	53.73 ± 60.60	0.003
Severity of legal problem	2.87 ± 1.27	2.43 ± 1.48	0.024
Family information			
Father’s alcoholism	17 (14.2)	29 (30.5)	0.004
Social information			
Physical abuse	24 (20.0)	9 (9.6)	0.038
Psychological information			
Serious depression	83 (69.2)	53 (55.8)	0.047
Trouble with controlling violent behavior	75 (62.5)	40 (42.1)	0.004
Prescription experience	55 (45.8)	26 (27.4)	0.007

The categorical variables are presented as *n* (%) in the chi-square test, and the continuous variables are presented as the mean ± SD in the Student’s *t* test.

### Analysis of the factors that affect the suicide attempts of substance users

Of the 215 subjects who had suicidal ideation, the factors that affected suicide attempts were analyzed in the 120 subjects who attempted suicide. In the logistic regression, the suicide attempts were found to have been associated with the protective supervision, which had an OR of 0.44, the father’s alcoholism (OR, 0.27), experiences of physical abuse (OR, 2.67), trouble with controlling violent behavior (OR, 2.02), and experiences of receiving doctors’ prescriptions due to psychological and emotional problems (OR, 2.24) (Table 4).

**Table 4:** Logistic stepwise regression analysis of substance user-attempted suicide

**Category**	**Odds ratio**	**95% CI**	***P *** **value**
Protective supervision	0.441	0.243–0.800	0.007
Father’s alcoholism	0.271	0.131–0.558	<0.001
Physical abuse	2.667	1.075–6.620	0.034
Trouble with controlling violent behavior	2.019	1.203–4.007	0.008
Prescription experience	2.244	1.257–4.007	0.006

## Discussion

Suicide is closely associated with psychiatric disorders. In particular, substance use has been confirmed to be associated with suicidal behavior in many aspects. Moreover, substance use disorders induce mood disorders as complications, increase treatment resistance, result in psychological trauma, and increase the risk of suicide attempts [7]. Therefore, it is very important to confirm the psychiatric and social factors that affect the type of substance use, the diagnosis of conduct disorders, and the possibility of suicidal behavior in the assessment of substance use disorder patients.

In this study, ASI was used as the most appropriate tool in the investigation of the seriousness of substance abuse and its related factors to confirm the factors associated with suicidal ideation and suicide attempts. The analysis of the seven domains of Medical Information, Employment, Drug/Alcohol Use, and Legal, Family, Social, and Psychological aspects had the following outcomes. The substance users who were not satisfied with their domestic environment during their growth, did not enjoy their spare time with others, and were not satisfied with their spare time experiences were confirmed to have been more likely to have had suicidal ideation. In addition, the subjects with more experiences of emotional abuse such as verbal abuse reported more instances of suicidal ideation. A more severe history of depression that included the feelings of sorrow, despair, loss of interest, restlessness, and guilt in addition to the difficulty of managing daily activities was found to have had a higher association with suicidal ideation. This result coincided with that of a recent study that reported the association of suicidal ideation with current depression and life problems in cases of substance use disorders [11,12].

The analysis of the factors of suicide attempts among substance users confirmed that less protective supervision resulted in more suicide attempts. In addition, past experiences of physical abuse caused more suicide attempts, and the subjects who had trouble with controlling their violent behavior were confirmed to have had more suicide attempts. In the psychiatric aspects, the subjects with experiences of receiving doctors’ prescriptions due to psychological and emotional problems reported more than two times more suicide attempts than the control group. This result coincided with the results of the study on the suicide attempts and deliberate self-harm of substance users, i.e., they are associated with childhood abuse as well as physical and sexual abuse, posttraumatic stress, and difficulties in controlling impulsive behavior when distressed [13]. However, unlike the study on suicide attempts, which reported emotional abuse as the only factor associated with suicide, this study confirmed that physical abuse caused OR 2.7 more suicide attempts.

Among the substance users in this study who planned to commit suicide with suicidal ideation, those whose father was an alcoholic had an OR 0.3 decrease in suicide attempts. This result was contrary to the authors’ initial expectations, but it may be explained as due to the substance user’s excessive sense of responsibility over his/her siblings in such a family. It is related to the hero on their childhood with respect to orientation of the codependency of alcoholic family.

The relationships between substance use disorders and completed suicide [14], and between alcohol/drug use disorders and nonfatal suicidal behavior [15] all confirmed that suicide risk is elevated in substance use disorders. Current reviews have identified some gaps that need to be filled for future studies, such as a comparison of attempted suicide and completed suicide, the proximal role of substance use as a trigger of suicidal behavior, the association between substances other than alcohol and suicidal behavior, the association between suicide and substance use disorders in special populations such as younger age groups, and the outcome of combinations of risk factors (e.g., substance use disorders and psychiatric morbidity) and suicidal behavior [16]. Furthermore, in treated samples from people with substance use disorders, more severe patterns of cocaine use [17] and sedative or benzodiazepine use [18-20], and more extensive polysubstance use [18,19,21] have all been linked to a greater likelihood of attempting suicide [22].

The risk of suicide in psychiatric patients is 3–12 times higher than in the general population [4]. The mood disorder associated with suicidal behavior in 15% of suicides was investigated. In addition, in schizophrenia, a 4% lifetime risk of suicide has been reported [3]. Accordingly, this study suggests that substance users’ rate of suicidal ideation and suicide attempts have significant implications.

The limitations of this study were as follows. First, the same-gender ratio could not be achieved among the subject substance users. Second, the types of substances that the subjects used were diverse, and some of them were used together, so the features of the drugs were not analyzed. In a future study on suicide-related behavior, an appropriate gender ratio of substance users and analyses by drug type may be needed.

## Conclusion

In this study, half of the substance users who planned to commit suicide actually attempted to commit suicide. Since the risk of committing suicide was confirmed to increase with suicidal ideation and planning, preventive measures such as policy establishment, education, and consultation may be needed. Various aspects of suicide-related behavior were analyzed that reflect the social seriousness of substance use in South Korea, and these multiple approaches are considered meaningful. Based on the outcomes of this study, action programs that involve suicide-prevention education and treatment may be prepared for substance users who have a high risk of committing suicide.

## Competing interests

The authors declare that they have no competing interests.

## Authors’ contributions

MK contributed to the design of the project, analysis of data, and writing of the paper. MK, SY, and KP contributed to theoretical interpretation and provided significant input on the manuscript. DJK contributed to the design of the project, supervision of data collection, and provision of significant input on the manuscript. All authors read and approved the final manuscript.
